# Exendin-4 ameliorates high glucose-induced fibrosis by inhibiting the secretion of miR-192 from injured renal tubular epithelial cells

**DOI:** 10.1038/s12276-018-0084-3

**Published:** 2018-05-01

**Authors:** Yijie Jia, Zongji Zheng, Meiping Guan, Qian Zhang, Yang Li, Ling Wang, Yaoming Xue

**Affiliations:** Department of Endocrinology & Metabolism, Nanfang Hospital, Southern Medical University, Guangzhou, China

## Abstract

Extracellular vesicles (EVs), which contain microRNA (miRNA), constitute a novel means of cell communication that may contribute to the inevitable expansion of renal fibrosis during diabetic kidney disease (DKD). Exendin-4 is effective for treating DKD through its action on GLP1R. However, the effect of exendin-4 on EV miRNA expression and renal cell communication during the development of DKD remains unknown. In this study, we found that EVs derived from HK-2 cells pre-treated with exendin-4 and high glucose (Ex-HG), which were taken up by normal HK-2 cells, resulted in decreased levels of FN and Col-I compared with EVs from HK-2 cells pre-treated with HG alone. Furthermore, we found that pretreatment with HG and exendin-4 may have contributed to a decrease in miR-192 in both HK-2 cells and EVs in a p53-dependent manner. Finally, we demonstrated that the amelioration of renal fibrosis by exendin-4 occurred through a miR-192-GLP1R pathway, indicating a new pathway by which exendin-4 regulates GLP1R. The results of this study suggest that exendin-4 inhibits the transfer of EV miR-192 from HG-induced renal tubular epithelial cells to normal cells, thus inhibiting GLP1R downregulation and protecting renal cells. This study reports a new mechanism by which exendin-4 exerts a protective effect against DKD.

## Diabetic kidney disease: Foiling cellular communication

An existing drug for diabetic kidney disease (DKD) helps limit the spread of the condition by preventing damaged cells from communicating with healthy cells. A key characteristic of DKD is renal fibrosis, a progressive thickening and scarring of connective tissues in the kidney exacerbated by high glucose levels. During fibrosis, injured cells release membrane-derived structures called extracellular vesicles (EVs) which infiltrate normal cells and help fibrosis spread. Now, Yaoming Xue and co-workers at the Southern Medical University in Guangzhou, China, have shown how an existing DKD drug, extendin-4, works to regulate EVs released from renal cells. Extendin-4 reduces the expression of a key microRNA molecule, miR-192, and this weakens EV-related cellular communication. The same mechanism also reduces the expression of an insulin-related protein known to be involved in the progression of DKD.

## Introduction

With the increase in the prevalence of diabetes mellitus, diabetic kidney disease (DKD) has become the leading cause of chronic kidney disease worldwide^1^. One of the most common characteristics of DKD is tubulointerstitial fibrosis, which accelerates renal failure and appears early in diabetic kidney injury^2, 3^. A previous study indicated that hyperglycemia can induce extracellular matrix accumulation of renal tubular epithelial cells, which is a vital step in tubulointerstitial fibrosis^4–6^.

Studies have reported that injured renal tubular epithelial cells can influence normal cells and other resident renal cells through the release of extracellular vesicles (EVs), resulting in a vicious cycle of renal fibrosis^7, 8^. EVs, which contain proteins, mRNA, and microRNA (miRNA), reflect a newly discovered method of cell-to-cell communication^9, 10^. Existing research indicates that EVs can distribute miRNA among cells, thereby promoting disease progression^11, 12^. However, the role of EV-mediated miRNA delivery in the progression of DKD remains unclear.

Exendin-4, a long-acting GLP-1 analog, has been used for the treatment of type 2 diabetes mellitus. GLP-1 exerts its biological action by binding to its specific receptor, the GLP-1 receptor (GLP1R), which is present in various organs, such as the liver, brain, and kidney^13, 14^. In addition to directly targeting GLP1R, exendin-4 has been indicated by many studies to potentially function through other mechanisms. Lee et al.^15^ reported that the levels of several miRNAs in the pancreas were altered after treatment with exendin-4, suggesting that exendin-4 may exert its function through miRNA; however, the mechanism remains unclear. p53, a transcription factor that promotes DKD progression^16^ and regulates several miRNAs, is reportedly downregulated by exendin-4^17^. Thus, we propose that exendin-4 may regulate miRNA expression through p53.

In this study, we aimed to examine the effects of exendin-4 on miRNA expression in renal tubular epithelial cells and in the EVs from these cells. We also determined whether exendin-4 influences EV miRNA delivery from high glucose (HG)-treated renal tubular epithelial cells to normal ones and determined the underlying mechanisms.

## Materials and methods

### Cell culture and treatment

The human renal tubular epithelial cell line HK-2 (ATCC, Manassas, USA) was cultured in Dulbecco’s modified Eagle’s medium with 5.6 mM glucose (NG) supplemented with 10% fetal bovine serum (FBS; Gibco, Australia). The cells were incubated in a 5% CO_2_ incubator at 37 °C. When HK-2 cells were seeded at ~60% confluence, they were cultured in 2% FBS DMEM for 24 h and subsequently exposed to DMEM-containing 30 mM glucose (HG) and exendin-4 (0, 0.1, 1, 10, or 100 nM) for an additional 48 h.

For cell transfection, cells were transfected with miR-192 mimic, miR-192 inhibitor or GLP1R siRNA, and the appropriate negative controls (Ribo, China) at a concentration of 50 nm, and seeded at 60% confluence using Lipofectamine 3000 (Invitrogen, CA, USA) according to the manufacturer’s protocol. For co-culture experiments, EVs isolated from donor cells were added to recipient cells at a concentration of 50 μg/ml.

Cells were harvested 48 h after transfection or co-culture.

### EV extraction

HK-2 cells were cultured in DMEM medium with 5.5 mM d-glucose and 10% FBS until they reached 60% confluence. Subsequently, the media was changed to DMEM with 5.5 mM d-glucose, 30 mM d-glucose, or 30 mM d-glucose with 10 nM exendin-4 or transfected with the miR-192 inhibitor in 2% EV-depleted FBS for 48 h. EVs were removed from FBS using a previously reported procedure^18^. The culture supernatants were collected and centrifuged at 3000×*g* for 15 min to remove cells and cell debris. Subsequently, one-third volume of ExoQuick-TC (System Biosciences) was added to the supernatants and mixed. After 24 h of refrigeration, the mixture was centrifuged at 1500×*g* for 30 min, and the supernatant was removed. EVs were suspended in 30 µl of PBS and were stored at −80 °C for further analysis.

### Transmission electron microscopy (TEM)

For TEM, 20 µl of a fresh EV sample was placed on a 200-mesh-nickel grid, excess fluid was removed, and a drop of 2% phosphotungstic acid was transferred to the grid for 5 min for staining. Samples were analyzed with a JEOL JEM-1400 transmission electron microscope.

### Fluorescent labeling of EVs

EVs (50 µg) suspended in 160 µl of PBS were incubated in 20 µl of 5 µg/ml Dil-C18 at room temperature for 30 min. EVs were washed with PBS and harvested by centrifugation (200,000×*g* for 1 h). Subsequently, EVs were resuspended in 5.5 mM d-glucose DMEM and incubated with recipient cells at a concentration of 50 μg/ml for 24 h. Next, the cells were washed twice, stained with DAPI, and observed under an Olympus microscope (Japan).

### RNA isolation and reverse transcription quantitative PCR

Total RNA was prepared from cells using TRIzol reagent (Takara, Dalian, China) according to the manufacturer’s instructions. RNA quality was measured using a NanoDrop ND-1000 spectrophotometer (Thermo Fisher Scientific, Wilmington, DE, USA).

Approximately 500 ng of RNA was used for cDNA synthesis with a Takara Prime Script® RT reagent kit (Takara, Dalian, China). SYBR Premix Ex Taq™ (Takara) was used to quantify mRNA expression, and β-actin was used as an internal control. Primers for GLP1R, fibronectin (FN), type I collagen (Col-I), and β-actin were synthesized by Invitrogen (Shanghai, China) as follows: GLP1R: forward 5′-TCAAGGTCAACGGCTTATTAGTGAA-3′, reverse 5′-CCCAAGTGATGCAAGCAGAG-3′; FN: forward 5′-TAGCCCTGTCCAGGAGTTCA-3′, reverse 5′-CTGCAAGCCTTCAATAGTCA-3′; Col-I: forward 5′-GCAGGAGGTTTCGGCTAAGT-3′, reverse 5′-GCAACAAAGTCCGCGTATCC-3′; and β-actin: forward 5′-CCCTGGACTTCGAGCAAGAGAT-3′, reverse 5′-GTTTTCTGCGCAAGTTAGG-3′.

EV miRNA was extracted using a miRcute miRNA isolation kit (TianGen, China). A miRcute miRNA first-strand cDNA synthesis kit (TianGen, China) was used for small RNA reverse transcription. Subsequently, a miRcute miRNA qPCR detection kit (TianGen, China) was used to quantify miRNA expression; U6 was used as an internal control. Primers for miR-192 and U6 were purchased from TIANGEN.

RT-PCR was performed using a LightCycler 480 Real-Time PCR System (Roche; Hoffmann-La Roche Ltd, Basel, Switzerland). The fold-change of each mRNA/miRNA was calculated using the 2^−△△Ct^ method.

### Western blotting

Total protein was extracted with RIPA lysis buffer (KeyGEN Biotech, China), and protein concentrations were measured using the BCA method (Takara Biotechnology, Japan). Subsequently, 50 μg of protein was separated on a 10% SDS-PAGE gel and transferred to a PVDF membrane (Merck Millipore, MA, USA). Membranes were blocked at room temperature for 1 h and then incubated at 4 °C overnight with primary antibodies for FN (Sigma, St. Louis, MO, USA), Col-I (Merck Millipore, Germany), GLP1R (Abclonol, USA), p53 (Cell Signaling, MA, USA), CD63 (Santa Cruz, CA, USA), and β-actin (Santa Cruz, CA, USA). After an additional incubation with a fluorescent secondary antibody at room temperature for 1 h (1:15,000; LI-COR Biosciences, Lincoln, NE, USA), images were visualized with an Odyssey infrared imaging system (LI-COR, Lincoln, NE, USA), and densitometry analysis was performed using Quantity One software (Bio-Rad, USA).

### Luciferase assay

To create the GLP1R 3′-UTR luciferase reporter plasmid, a pMIR-REPORT vector (Ambion, USA) was used, and nucleotides 262–461 of the GLP1R 3′-UTR were cloned into the plasmid. A similar plasmid with a mutated miR-192 seed sequence (from AGGTCAA to CATGTGC) was also constructed using a site-directed mutagenesis kit (TianGen, China).

HK-2 cells were seeded in a 24-well plate and transfected with a mixture of 1 µg of pMIR-REPORT plasmid, 0.2 µg of β-gal plasmid (Ambion, USA), and 50 nmol miR-192 or negative control (NC) mimic and inhibitor with Lipofectamine 3000 (Invitrogen, CA, USA). The cells were lysed 48 h after transfection, and luciferase activity was measured with a luciferase assay kit (Beyotime, China). All experiments were repeated three times.

### Chromatin Immunoprecipitation (CHIP) analysis

A ChIP-IT^®^ Express kit (Active Motif, Bedford, MA) was used to perform the ChIP assay. HK-2 cells were treated with NG, HG or HG + exendin-4 for 48 h and cross-linked using 1% formaldehyde in DMEM; the crosslink reaction was stopped by glycine. Samples were collected, resuspended, and sheared to 800 bp. Subsequently, 2 µg anti-p53 and 2 µg IgG antibody (Cell Signaling, MA, USA) was used for immunoprecipitation. Input DNA was evaluated and subjected to RT-PCR according to the manufacturer’s instructions. Briefly, a standard curve was made by performing qPCR with a primer targeting known DNA quantities of input DNA. Subsequently, ChIP Ct and IgG Ct values were used to calculate the ChIP DNA and IgG quantity. Fold enrichment was calculated as ChIP DNA quantity/IgG DNA quantity. Primers for the p53-binding sites on the miR-192 promoter region were designed according to a previous manuscript^19^.

### EV exendin-4 dosage by high-performance liquid chromatography (HPLC)

EVs were pre-treated for HPLC measurement using the methods reported by Pascucci et al.^20^ Briefly, EV samples were added to equal volumes of 0.6 N perchloric acid, mixed and centrifuged at 200×*g* at 4 °C for 10 min, followed by filtration and injection into HPLC. An Agilent 1260 system combined with diode-array detection (DAD) was used for HPLC analysis of these samples. An Agilent C18 column (5 µm, 250 mm × 4.6 mm) with an HPLC guard cartridge system (Phenomenex, SecurityGuard) was used for HPLC analysis. The mobile phases consisted of acetonitrile (A) and 20 mmol/l KH_2_PO_4_ with pH adjusted to 3 with phosphoric acid, (B) using an isocratic elution program of 37% A for 0–30 min. The flow rate was 1. 0 ml/min, and column temperature was set to 30 °C. The DAD detector was set at 210 nm.

### Statistical analyses

All data are presented as the mean ± SEM. Student’s *t*-test and one-way ANOVA were used to determine statistical significance. *P*-values < 0.05 were considered statistically significant.

## Results

### Exendin-4 alleviates HG-induced fibrosis in HK-2 cells

**Fig. 1:**
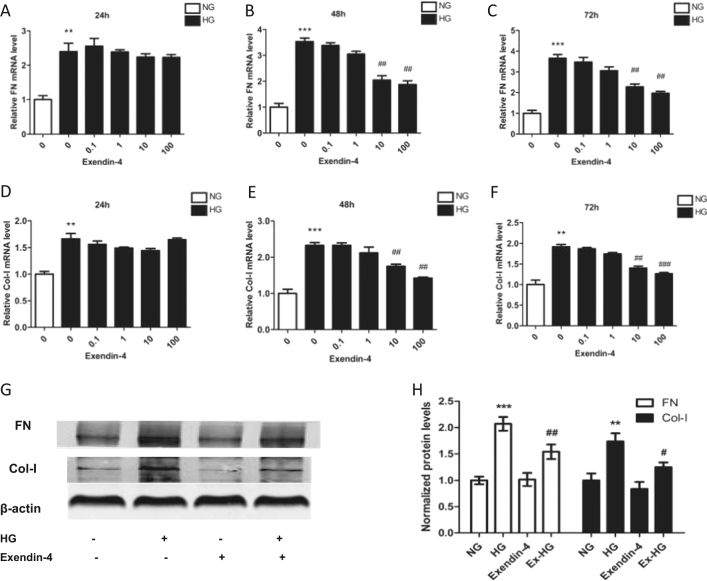
Effect of exendin-4 on fibrosis in HG-induced HK-2 cells. HK-2 cells were cultured in DMEM with 5.6 mM glucose (NG) or 30 mM glucose (HG) and different concentrations of exendin-4. **a**–**f** Relative expression of FN and Col-I in HK-2 cells after incubation with HG and various concentrations of exendin-4 for 24, 48, and 72 h. **g**, **h** FN and Col-I expression in HK-2 cells after incubation with HG and 10 nM exendin-4 for 48 h was analyzed by western blot (**P < 0.01 and ***P < 0.001 versus the NG group; ^#^*P* < 0.05,^##^*P* < 0.01, and ^###^*P* < 0.001 versus the HG group)

To determine the effects of exendin-4 on HK-2 cell fibrosis under HG conditions, we treated HK-2 cells with various concentrations of exendin-4 for 24, 48, or 72 h. Treatment with HG significantly increased the levels of FN and Col-I mRNA, whereas large doses of exendin-4 (10 and 100 nM) decreased FN and Col-I expression (Fig. 1a–f). In addition, treatment with 10 nM exendin-4 for 48 h also decreased HG-induced FN and Col-I expression at the protein level (Fig. 1g, h)

### Exendin-4 reverses the production of EVs induced by HG treatment

**Fig. 2:**
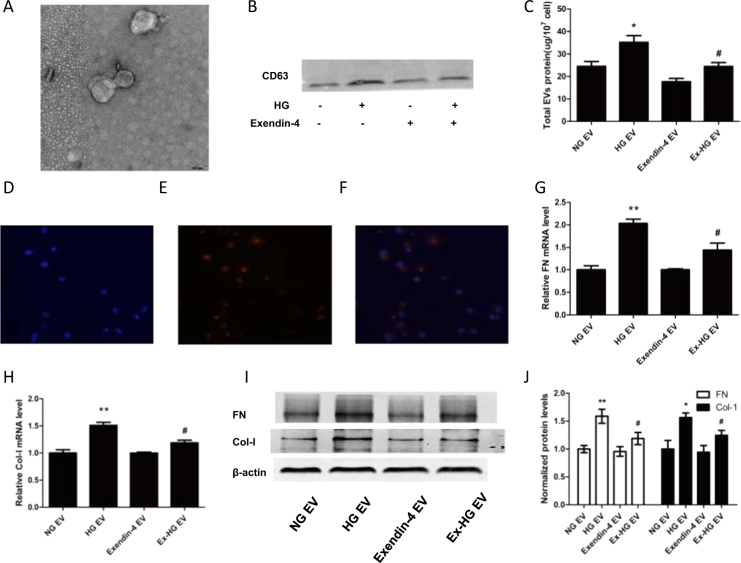
Treatment of HK-2 cells with Ex-HG group-derived EVs induced less fibrosis in recipient cells than treatment with HG group-derived EVs. **a** EVs from HK-2 cells identified using TEM. **b** Western blot of CD63 expression of in EVs from 5 × 10^7^ cells. **c** Total EV protein from the NG group, HG group, exendin-4 group, and Ex-HG group. **d**–**f** Co-culture of EVs with recipient cells. EVs from donor cells were labeled with Dil-C18, and recipient cells were labeled with DAPI; red represents Dil-C18, and blue represents nuclear DNA. **g**,** h** Relative expression of FN and Col-I in HK-2 cells after incubation with EVs derived from NG, HG, exendin-4, and Ex-HG groups for 48 h. **i**, **j** The FN and Col-I expression in HK-2 cells after incubation with EVs derived from NG, HG, exendin-4, and Ex-HG groups for 48 h was analyzed by western blot (*P < 0.05 and **P < 0.01 versus the NG EV group; ^#^*P* < 0.05 versus the HG EV group)

To explore the role of HG and exendin-4 on EV release, we first isolated EVs from renal tubular epithelial cells. TEM revealed that EVs appeared as a cluster of cup-shaped vesicles (Fig. 2a), and immunoblotting confirmed the expression of CD63 (Fig. 2b).

To explore the effect of exendin-4 on EV release, we used total EV protein/total cell number to evaluate the production of EVs. The HG group had a significantly greater total number of EVs than the control group. The addition of exendin-4 to the HG group (Ex-HG) significantly reversed EV production (Fig. 2c).

### EVs derived from exendin-4 and HG-pre-treated (Ex-HG) donor cells do not promote fibrosis in recipient cells

EVs from injured renal tubular epithelial cells have been reported to promote the fibrosis in recipient cells^8^. Because exendin-4 alleviates HG-induced fibrosis, we examined whether EVs from Ex-HG-treated donor cells led to fibrosis in recipient cells. To confirm that EVs can be transported into recipient cells, we labeled EVs with Dil-C18. After 24 h of incubation, Dil-C18 appeared in the cytoplasm of recipient cells (Fig. 2d–f), which indicated that EVs were transported into the recipient cells.

Furthermore, EVs from the NG, HG, exendin-4, and Ex-HG groups were collected and co-cultured with HK-2 cells. As expected, the EVs derived from the HG group markedly increased the levels of FN and Col-I, but the addition of exendin-4 to donor cells (Ex-HG) resulted in decreased levels of FN and Col-I in recipient cells compared with HG group-derived EVs (Fig. 2g–j).

**Fig. 3:**
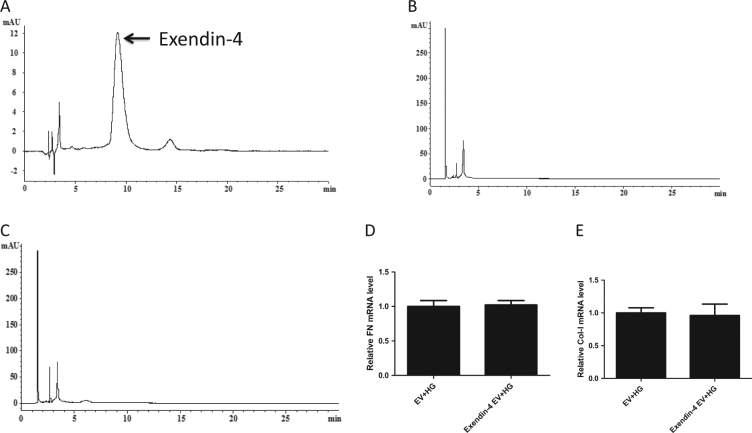
Exendin-4 group-derived EVs loaded little exendin-4. **a** The HPLC chromatogram from the standard sample of exendin-4 in PBS (1 mg/ml). **b** The HPLC chromatogram from NG group-derived EVs. **c** The HPLC chromatogram from exendin-4 group-derived EVs. **d**, **e** Relative expression of Col-I and FN in HG-treated HK-2 cells co-cultured with exendin-4 group-derived EVs

To determine whether the ability of EVs from the Ex-HG group to alleviate the fibrosis effect on HK-2 cells was mediated by exendin-4 within the EVs, we measured the concentration of exendin-4 in EVs derived from exendin-4-treated HK-2 using HPLC. As shown in Fig. 3a–c, the HPLC chromatograms from EVs derived from exendin-4-treated HK-2 cells did not exhibit a peak at the identical retention time as the peak from the standard sample of exendin-4 in PBS (1 mg/ml), suggesting that little exendin-4 was packaged into EVs from the exendin-4 group. Further, after we co-cultured HG-treated HK-2 cells with EVs from exendin-4-treated HK-2 cells, we did not observe changes in FN or Col-I expression (Fig. 3d, e).

### Exendin-4 downregulates HG-induced miR-192 expression in EVs

**Fig. 4:**
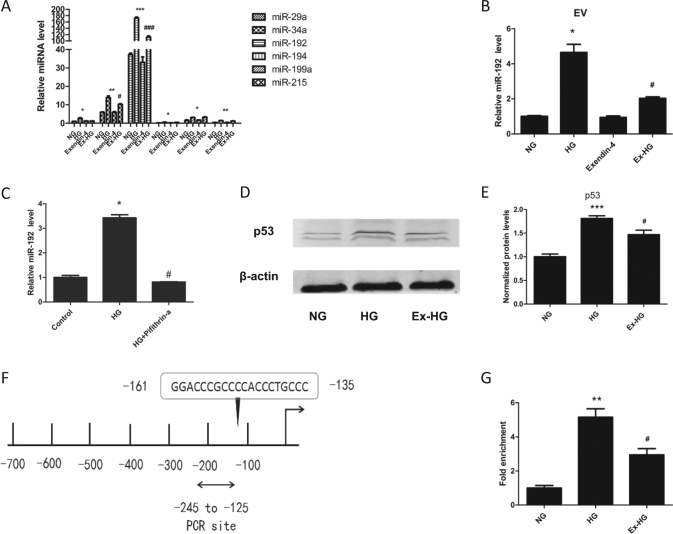
Exendin-4 inhibited miR-192 expression via p53. **a** Effect of exendin-4 treatment on the expression of miRNA in HK-2 cells exposed to HG. **b** Effect of exendin-4 treatment on the expression miR-192 in EVs derived from HK-2 cells exposed to HG. **c** Effect of pifithrin-α treatment on the expression miR-192 in HK-2 cells exposed to HG. **d**, **e** Effect of exendin-4 treatment on the expression of p53. **f** Predicted p53-binding sites on the miR-192 promoter. **g** Exendin-4 inhibits the binding of p53 to the miR-192 promoter (*P < 0.05, **P < 0.01, and ***P < 0.001 versus the NG group; ^#^*P* < 0.05 versus the HG group)

miRNA has been reported to be important in EV-mediated cell-to-cell communication. Thus, we proposed that the EVs derived from the Ex-HG group alleviated the fibrosis effect on HK-2 cells through miRNA. p53 has a vital role in regulating miRNA expression, and the miR-192 family, miR-29a, miR-34a, and miR-199, are controlled by p53 and have a role in renal fibrosis^16, 21^. Therefore, we analyzed the expression of these miRNAs in HG-stimulated and exendin-4-stimulated HK-2 cells, and determined that miR-192 was most significantly increased after HG treatment and repressed by exendin-4 (Fig. 4a). Furthermore, as shown in Fig. 4b, the expression of miR-192 was significantly downregulated in Ex-HG group-derived EVs compared with that in HG group-derived EVs. To confirm that exendin-4 regulated miR-192 expression through p53, we first used pifithrin-a to inhibit p53 expression. As shown in Fig. 4c, the expression of miR-192 was significantly downregulated in HG + pifithrin-a-treated HK-2 cells, indicating that HG regulated miR-192 expression in a p53-dependent manner. We then investigated the underlying mechanism by which exendin-4 regulates miR-192. As shown in Fig. 4d and e, HG upregulated p53 expression, but this increase was reversed by exendin-4. Using a ChIP assay, we determined that HG promoted p53 binding to the promoter of miR-192, which was blocked by exendin-4 (Fig. 4f, g).

### Exendin-4 downregulates miR-192 expression induced by HG in recipient cells

**Fig. 5:**
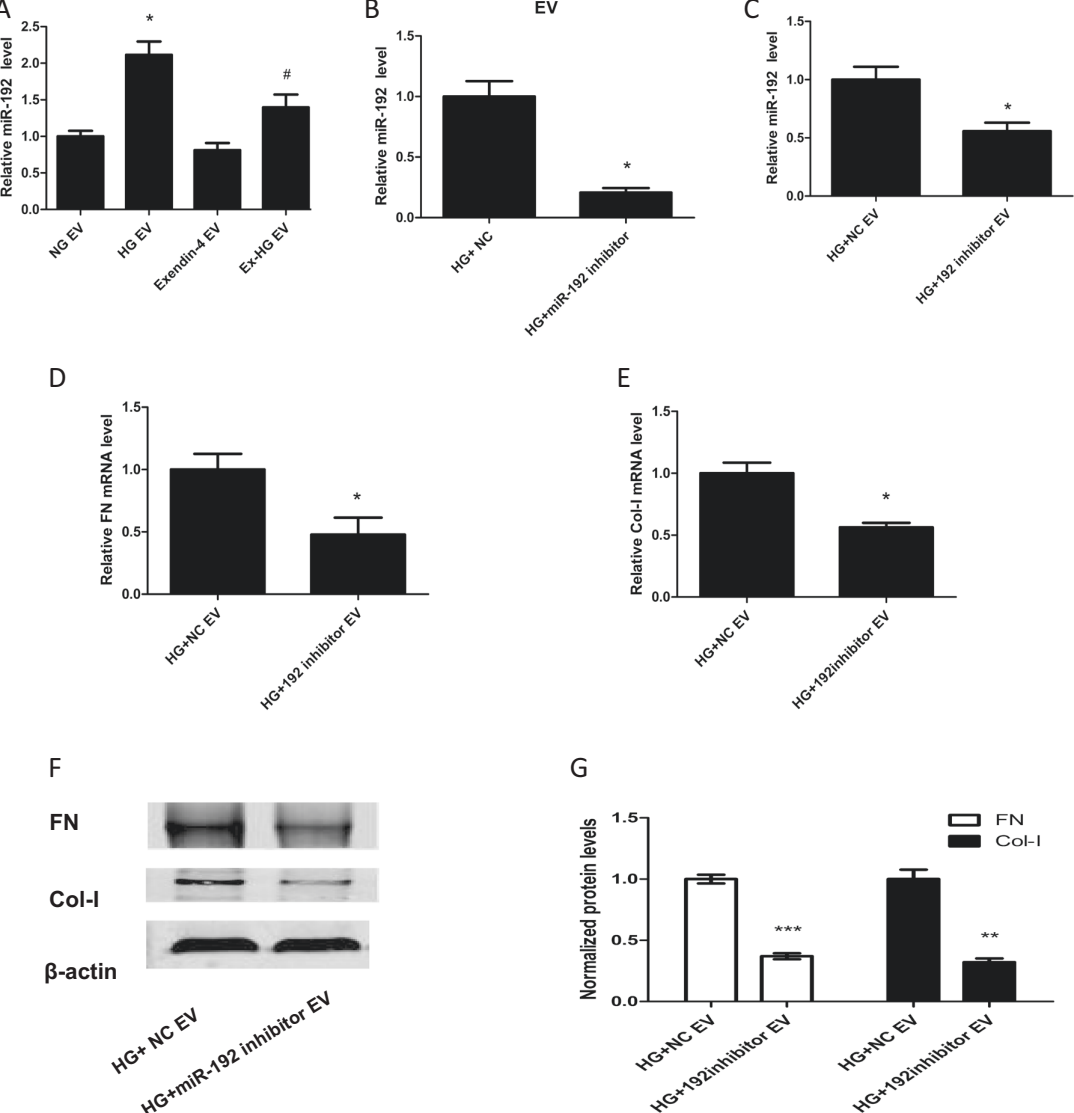
miR-192 in donor-cell EVs induced fibrosis in recipient cells. **a** Relative expression of miR-192 in HK-2 cells after incubation with EVs derived from NG, HG, exendin-4, and Ex-HG groups for 48 h (*P < 0.05 versus the NG EV group; ^#^*P* < 0.05 versus the HG EV group). **b** Relative expression of miR-192 in EVs derived from HK-2 cells pre-transfected with the miR-192 inhibitor or negative control (NC) and exposed to HG for 48 h (*P < 0.05 versus the HG + NC group). **c**–**e** Relative expression of miR-192, Col-I, and FN in HK-2 cells after incubation with EVs derived from HK-2 cells pre-transfected with the miR-192 inhibitor or NC and exposed to HG for 48 h. **f**, **g** Col-I and FN expression in HK-2 cells after incubation with EVs derived from HK-2 cells pre-transfected with the miR-192 inhibitor or NC and exposed to HG for 48 h was analyzed by western blot (*P < 0.05 and ***P < 0.001 versus the HG + NC EV group)

To determine whether miR-192 can be transported through EVs, EVs from the NG, HG, exendin-4, and Ex-HG groups were collected and co-cultured with HK-2 cells. As shown in Fig. 5a, miR-192 levels in recipient cells co-cultured with EVs from HG-treated cells were significantly upregulated compared with the levels in cells co-cultured with EVs from NG-treated cells, whereas levels were reduced in cells co-cultured with EVs from Ex-HG-treated cells compared with the levels in those treated with HG group-derived EVs. However, we did not observe a change in miR-192 levels in recipient cells co-cultured with EVs from the exendin-4 group compared with the levels in those co-cultured with EVs from the NG group.

### EV-mediated miR-192 delivery into recipient cells promotes fibrosis

To further explore the role of EV miR-192 in the promotion of renal fibrosis, we transfected HK-2 cells with a miR-192 inhibitor before HG treatment, collected the EVs from these cells, and co-cultured the EVs with recipient cells. As shown in Fig. 5b, the miR-192 inhibitor significantly downregulated miR-192 expression in EVs, and these EVs did not upregulate miR-192 expression in recipient cells (Fig. 5c). Furthermore, we analyzed FN and Col-1 expression in recipient cells and found that the miR-192 inhibitor group-derived EVs blocked FN and Col-I upregulation (Fig. 5d–g). Thus, the fibrosis caused by the EVs derived from HG-treated cells may be induced by miR-192.

### miR-192 targets GLP1R in HK-2 cells

**Fig. 6:**
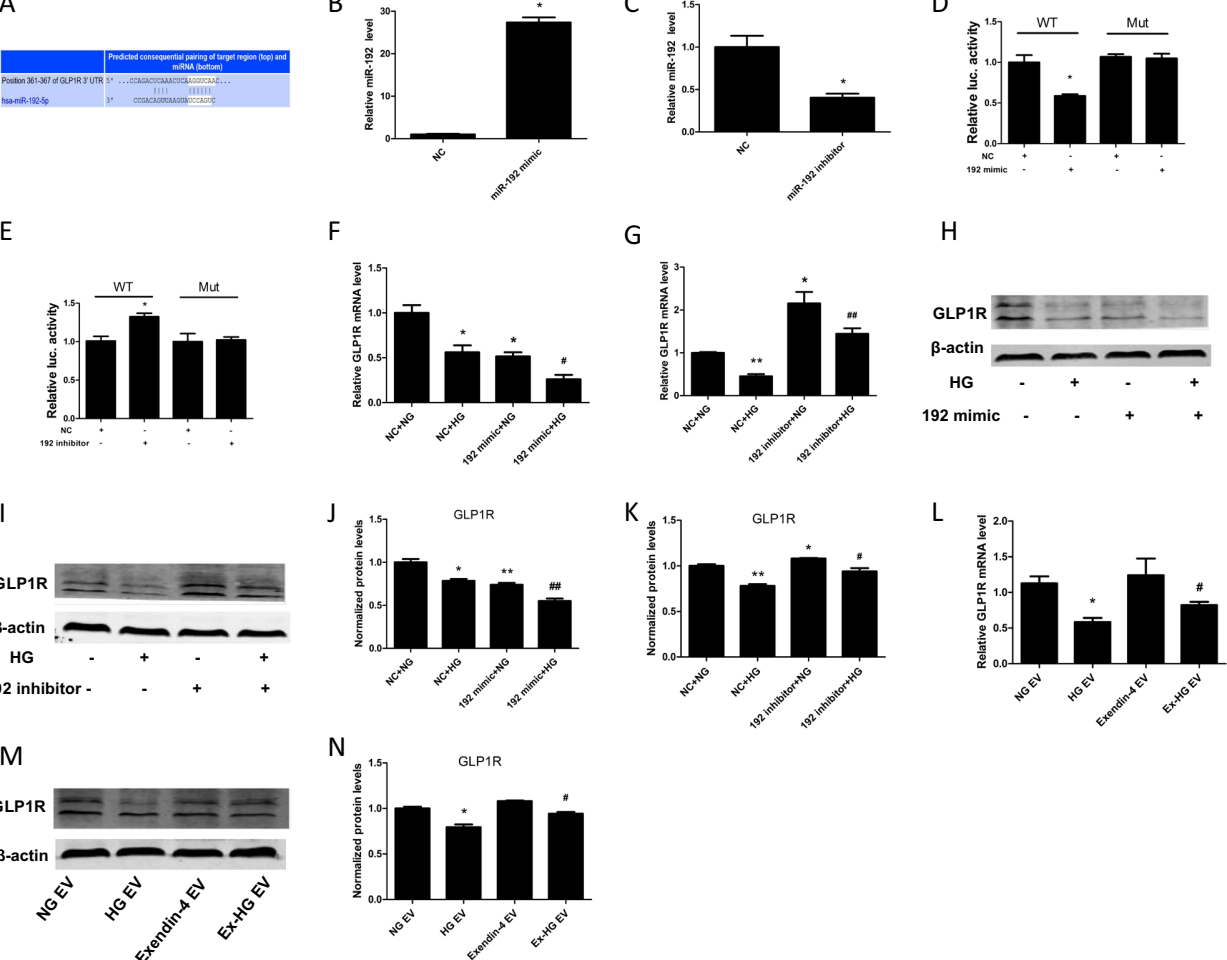
MiR-192 regulated GLP1R expression. **a** The predicted miR-192-binding sites within the 3′-UTRs of GLP1R. (**b**,**c**) Relative expression of miR-192 in HK-2 cells transfected with miR-192 mimic or inhibitor. **d**, **e** Relative luciferase activity in HK-2 cells co-transfected with miR-192 mimic, inhibitor, or NC and pMIR-REPORT containing the GLP1R 3′-UTR site (*P < 0.05 versus the NC group). **f**, **g** Relative expression of GLP1R in HK-2 cells transfected with miR-192 mimic or inhibitor and exposed to HG (*P < 0.05 and **P < 0.01 versus the NC + NG group; ^#^*P* < 0.05 and ^##^*P* < 0.01 versus the NC + HG group). **h**–**k** GLP1R expression in HK-2 cells transfected with miR-192 mimic or inhibitor and exposed to HG was analyzed by western blot (*P < 0.05 and **P < 0.01 versus the NC + NG group; ^#^*P* < 0.05 and ^##^*P* < 0.01 versus the NC + HG group). **l**–**n** Relative expression of GLP1R in HK-2 cells after incubation with EVs derived from NG, HG, exendin-4, and Ex-HG groups for 48 h (*P < 0.05 versus the NG EV group; ^#^*P* < 0.05 versus the HG EV group)

To further explore the mechanism by which miR-192 regulates renal fibrosis, we used TargetScan to identify miR-192 target genes. Interestingly, GLP1R, a receptor of GLP-1, was identified as a target of miR-192 (Fig. 6a). We explored whether GLP1R is a target of miR-192. First, we transfected HK-2 cells with a miR-192 mimic and miR-192 inhibitor to overexpress and downregulate miR-192 expression, respectively (Fig. 6b, c). On the basis of a luciferase reporter assay, the miR-192 mimic significantly decreased the 3ʹ-UTR activity of GLP1R (Fig. 6d), whereas the miR-192 inhibitor increased the 3ʹ-UTR activity of GLP1R (Fig. 6e). In addition, the miR-192 mimic aggravated the HG-induced decrease in the expression of GLP1R, whereas the miR-192 inhibitor reversed HG-induced GLP1R expression (Fig. 6f–k). Furthermore, EVs from HG-treated HK-2 cells also reduced GLP1R expression, and this effect was reversed by the EVs derived from the Ex-HG-treated cells (Fig. 6l–n).

### GLP1R knockdown induces renal fibrosis

**Fig. 7:**
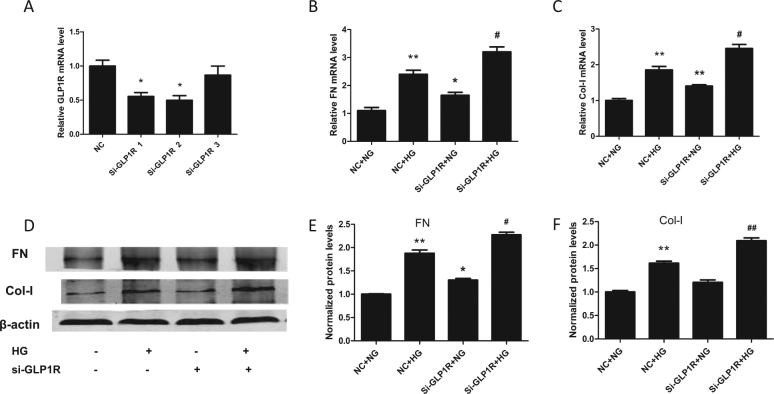
GLP1R knockdown induced renal fibrosis. **a** The silencing ability of three si-GLP1R sequences in HK-2 cells (*P < 0.05 versus the NC group). **b**, **c** Relative expression of Col-I and FN in HK-2 cells transfected with si-GLP1R and exposed to HG. **d**–**f** Col-I and FN expression in HK-2 cells transfected with si-GLP1R and exposed to HG was analyzed by western blot (*P < 0.05 and **P < 0.01 versus the NC + NG group; ^#^*P* < 0.05 and ^##^*P* < 0.01 versus the NC + HG group)

To evaluate the role of GLP1R in renal fibrosis, we transfected HK-2 cells with si-GLP1R. As shown in Fig. 7a, si-GLP1R-2 efficiently reduced GLP1R expression in HK-2 cells. Moreover, we evaluated the role of GLP1R in renal fibrosis and observed that si-GLP1R significantly upregulated FN and Col-I at both the mRNA and protein levels, which indicated that GLP1R has a role in renal fibrosis (Fig. 7b–f).

### miR-192 regulates renal fibrosis through GLP1R

**Fig. 8:**
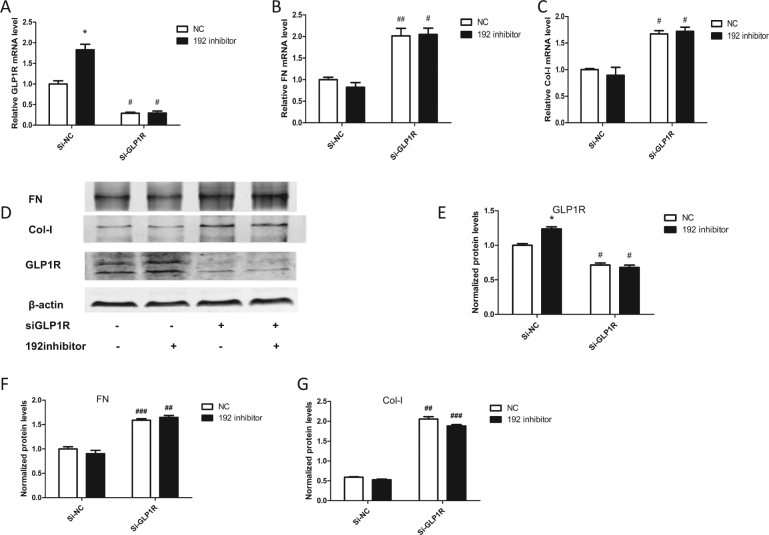
MiR-192 regulated renal fibrosis through GLP1R. **a**–**c** Relative expression of GLP1R, FN, and Col-I in HK-2 cells co-transfected with both si-GLP1R and the miR-192 inhibitor. **d**–**g** GLP1R, FN, and Col-I expression in HK-2 cells co-transfected with both si-GLP1R and the miR-192 inhibitor was analyzed by western blot (*P < 0.05 and **P < 0.01 versus the NC group; ^#^*P* < 0.05, ^##^*P* < 0.01, and ^###^*P* < 0.001 versus the Si-NC group)

To explore whether miR-192 promotes renal fibrosis through GLP1R, we co-transfected HK-2 cells with both si-GLP1R and a miR-192 inhibitor. As shown in Fig. 8a, the miR-192 inhibitor-induced GLP1R upregulation was blocked by si-GLP1R. As shown in Fig. 8b–g, amelioration of the renal fibrosis caused by the miR-192 inhibitor was abolished by si-GLP1R. These results indicate that miR-192 aggravates renal fibrosis through a GLP1R-dependent manner.

## Discussion

**Fig. 9:**
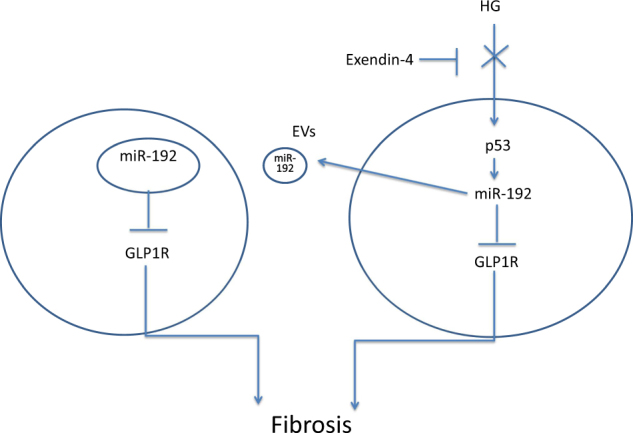
Exendin-4 inhibited the transfer of miR-192 and protected renal cells. Exendin-4 inhibited the transfer of EV miR-192 from HG-induced renal tubular epithelial cells to normal cells in a p53-dependent manner, thus inhibiting GLP1R downregulation by miR-192 and protecting renal cells

Exendin-4 has been shown to be beneficial for renal function by targeting GLP1R. In our study, we revealed a new mechanism by which exendin-4 can inhibit renal fibrosis. Specifically, exendin-4 downregulates cellular and secreted miR-192 expression induced by HG, thus inhibiting GLP1R downregulation in both donor and recipient cells (Fig. 9).

Recent studies have reported that exendin-4 exerts a renal-protective role independent of blood glucose control. In a diabetic mouse model, administration of exendin-4 decreased urinary albumin and ameliorated glomerular hypertrophy without changing blood glucose levels^22^. In our previous research, exendin-4 inhibited MC proliferation and FN secretion through AMPK activation^13^. In other research, exendin-4 exerted a protective renal function by restoring nitric oxide production and renal blood flow^23^. In addition, exendin-4 also improved renal function through activation of protein kinase A^24^. However, the effect of exendin-4 on renal miRNA expression has not been previously studied. In recent years, studies have suggested that some medications can exert an anti-disease effect by regulating miRNA expression. Arunachalam et al.^25^ reported that metformin can downregulate microRNA-34a to prevent diabetic vascular disease. Atrasentan was found to prevent miR-199 expression, thereby preventing renal tubular injury^26^. Transcription factors are known to have a role in regulating miRNA expression^27^. Zhao et al.^17^ found that exendin-4 attenuated p53 expression after 3 days of treatment. As a transcription factor, p53 can regulate several miRNAs^28^. After analysis, we found that miR-192 was most significantly increased after HG treatment and repressed by exendin-4 (Fig. 4a). To verify that HG regulates miR-192 expression in a p53-dependent manner, we treated cells with pifithrin-a, a p53 inhibitor, and HG and observed that the expression of miR-192 was significantly downregulated relative to the expression in HG-treated HK-2 cells. Furthermore, we observed that exendin-4 reversed HG-induced p53 expression (Fig. 4d, e). To explore the underlying mechanism, we used a ChIP assay to determine that HG promoted the binding of p53 to the promoter of miR-192 and that this effect was decreased by exendin-4 (Fig. 4f, g). These results indicate that exendin-4 regulates miR-192 expression during high-glucose treatment in a p53-dependent manner.

EVs have been reported to transfer information between cells and contribute to the development of disease. Borges et al.^7^ determined that exosomes released by injured epithelial cells carry TGF-β1 mRNA, and promote proliferation and fibrosis in fibroblasts. Zhou et al.^8^ reported that TGF-β-treated tubular epithelial cells promote renal fibrosis by transferring miR-21 to recipient tubular epithelial cells via EVs. These studies proved that EVs can transport damage signals. Therefore, we sought to determine whether exendin-4 can reduce the ability of injured renal tubular cells to transport damage signals to normal cells. First, we used Dil-C18-labeled EVs to confirm that the EVs entered HK-2 cells (Fig. 2d–f). Next, we measured FN and Col-I expression after co-culture with EVs and determined that HG-treated HK-2 cell-derived EVs induced normal HK-2 cell fibrosis. However, Ex-HG group-derived EV treatment prevented fibrosis compared with HG group-derived EV treatment (Fig. 2g–j), leading us to conclude that exendin-4 weakens the transmission of this damage signal. Previous research has proven that many factors can affect EV release, and HG has been shown to increase the release of EVs^29, 30^. Our research demonstrated that HG increased the total number of EVs but that Ex-HG decreased EV production compared with HG alone (Fig. 2c). Thus, exendin-4 may exert its effect by reducing the number of EVs.

EVs have been suggested to potentially be able to function as medication carriers. In one study, MSCs were shown to be able to package and deliver paclitaxel through MVs^20^. To explore whether the alleviated fibrosis effect of EVs from the Ex-HG group on HK-2 cells was mediated by exendin-4 contained in EVs, we measured the concentration of exendin-4 in EVs derived from exendin-4-treated HK-2 cells using HPLC. However, we determined that exendin-4 was not packaged in EVs, and EVs derived from exendin-4-treated HK-2 cells were unable to exert anti-fibrosis activity on HG-treated HK-2 cells. These observations indicate that little exendin-4 was loaded into EVs derived from exendin-4-treated HK-2 cells. In recent years, several studies have reported that directly loading EVs, instead of loading EVs via the cell of origin, is much more effective. Active loading, in which electric pulses are used to increase the permeability of the EVs, is more effective than merely incubating the isolated EVs with the therapeutic agent^31^. Thus, active loading may be a more effective method for the delivery of exendin-4 through EVs in future studies.

As miRNAs are reported to have a role in EV-mediated communication, we further explored the mechanism by which exendin-4 exerts its effect through EVs. We have reported that HG upregulates miR-192 expression in HK-2 cell-derived EVs^18^. In the present study, we observed that miR-192 levels were downregulated in Ex-HG group-derived EVs compared with those in HG group-derived EVs (Fig. 4b). To explore the role of miR-192, we used a miR-192 inhibitor to decrease miR-192 expression in EVs and observed that the EVs derived from miR-192 inhibitor-treated cells decreased FN and Col-1 expression compared with EVs from the HG group (Fig. 5c–g). Thus, our findings indicate that the EVs derived from HG-treated HK-2 cells induced renal fibrosis through the delivery of miR-192. Exendin-4 can decrease the levels of miR-192 in EVs, thereby blocking the transmission of signals from injured cells to normal cells. However, we observed that treatment of HK-2 cells with exendin-4 was unable to downregulate miR-192 expression compared with control treatment (Fig. 4a). Furthermore, we did not observe differences in EV miR-192 expression between the control group and the exendin-4 group (Fig. 4b). As shown in Fig. 5a, we observed no significant difference in miR-192 expression between HK-2 cells co-cultured with EVs from the NG group and those co-cultured with EVs from the exendin-4 group. These results indicate that exendin-4 significantly reduces miR-192 expression under HG conditions but has limited effectiveness under untreated conditions. Indeed, the role of exendin-4 under untreated conditions differs from cell to cell. In Nagayama’s study^32^, exendin-4 decreased ERK1/2 and JNK activation only under AngII stimulation. Zhao et al.^17^ also reported that exendin-4 was unable to downregulate p53 expression under untreated conditions. However, Wei et al.^33^ reported that exendin-4 exerts direct protective effects on endothelial cells through the AMPK/Akt/eNOS pathway. Thus, further study is needed to explore the mechanism by which exendin-4 exerts its protective effects.

Although miR-192 expression during DKD development remains controversial^34^, using TargetScan, we found that miR-192 may target GLP1R to aggravate renal fibrosis (Fig. 6a). We showed that miR-192 targets GLP1R by binding to its 3′-UTR and that overexpression of miR-192 reduced GLP1R levels (Fig. 6b–k). To verify that miR-192 promotes renal fibrosis through GLP1R, we co-transfected HK-2 cells with both si-GLP1R and a miR-192 inhibitor. The results showed that the amelioration of renal fibrosis caused by the miR-192 inhibitor was abolished by si-GLP1R (Fig. 8a–g). Thus, GLP1R is a key factor in the regulation of fibrosis by miR-192. However, in this study, we found that miR-192 altered both GLP1R protein and mRNA levels. Furthermore, the effect of miR-192 on mRNA transcription was greater than that on its protein levels. Therefore, miR-192 may exert its effects not only by directly targeting GLP1R but also through some other indirect mechanisms. One possible mechanism is that miR-192 could directly target SLC39A6 (Zip6)^35^, which has been identified as a putative GLP1R-interacting protein^36^. Alternatively, miR-192 may positively interact with TGF-β^37^, which could downregulate the AMPK pathway and thereby decrease GLP1R expression^38^.

GLP1R is highly expressed in the kidney, especially in renal tubular epithelial cells^39^. The development of DKD is accompanied by decreased GLP1R levels, and exposure of renal cells to HG or lipotoxicity reduces GLP1R expression^40^. Furthermore, reduced GLP1R expression leads to renal cell autophagy^39^ and apoptosis^38^. Fibrosis is crucial in the development of DKD progression; however, the role of GLP1R in HK-2 cell fibrosis is unclear. As previously reported, HG significantly reduced GLP1R expression in HK-2 cells^39^. We also showed that knockdown of GLP1R induced FN and Col-1 expression (Fig. 7b–f). These results indicate that GLP1R loss not only leads to renal cell autophagy and apoptosis but also promotes fibrosis, thus accelerating DKD progression. GLP1R agonists have been reported to protect renal function via inhibition of NAD(P)H oxidase and cAMP-PKA pathway activation^41^. However, in this study, we did not examine the mechanism by which GLP1R regulates renal fibrosis.

In conclusion, this research provides evidence that exendin-4 can inhibit the transfer of miR-192 from HG-induced renal tubular epithelial cells to normal cells, thus reversing GLP1R expression and protecting renal cells. These findings provide novel insight into the mechanism by which exendin-4 may be used to treat diabetic nephropathy.
